# Bone Regeneration Using N-Methyl-2-pyrrolidone as an Enhancer for Recombinant Human Bone Morphogenetic Protein-2 in a Rabbit Sinus Augmentation Model

**DOI:** 10.1155/2017/4153073

**Published:** 2017-06-07

**Authors:** Hyun-Chang Lim, Daniel S. Thoma, So-Ra Yoon, Jae-Kook Cha, Jung-Seok Lee, Ui-Won Jung

**Affiliations:** ^1^Department of Periodontology, School of Dentistry, Kyung Hee University, Seoul, Republic of Korea; ^2^Clinic for Fixed and Removable Prosthodontics and Dental Material Science, University of Zurich, Zurich, Switzerland; ^3^Department of Periodontology, Research Institute for Periodontal Regeneration, College of Dentistry, Yonsei University, Seoul, Republic of Korea

## Abstract

The aim of this study was to determine whether N-methyl-2-pyrrolidone (NMP) can decrease the dose of recombinant human bone morphogenetic protein-2 (rhBMP-2) in sinus augmentation of rabbits. In each of 15 rabbits, 2 sinuses were randomly grafted using 1 of 3 treatment modalities: (i) biphasic calcium phosphate (BCP; control), (ii) rhBMP-2-coated BCP (BMP), or (iii) rhBMP-2-coated BCP soaked in NMP solution (BMP/NMP). The rabbits were sacrificed 2 weeks postoperatively. Histologic and histomorphometric analyses were performed. Bone formation in all groups was predominantly located close to the access window and the lateral walls. Newly formed bone within the total augmented area (NB_TA_) was greatest in BMP/NMP (1.94 ± 0.69 mm^2^), followed by BMP (1.50 ± 0.72 mm^2^) and BCP (1.28 ± 0.52 mm^2^) (*P* > 0.05). In the center of the augmentation (NB_ROI_C_) and the area close to the sinus membrane (NB_ROI_M_), BMP/NMP produced the largest area of NB (NB_ROI_C_: 0.10 ± 0.11 mm^2^; NB_ROI_M_: 0.17 ± 0.08 mm^2^); the corresponding NB values for BCP were 0.05 ± 0.05 mm^2^ and 0.08 ± 0.09 mm^2^, respectively (*P* > 0.05 for all comparisons). The effect of NMP on bone regeneration was inconsistent between the specimens. Adding NMP as an adjunct to rhBMP-2-coated BCP produced inconsistent effects on bone regeneration, resulting in no significant benefit compared to controls.

## 1. Introduction

The challenges in implant dentistry include accomplishing predictable regenerative outcomes in severely damaged or atrophied sites within a short healing period. Conventional approaches often require a relatively high level of surgical skill to produce successful outcomes and are associated with increased patient morbidity [1]. The introduction of recombinant human bone morphogenetic protein-2 (rhBMP-2) was considered revolutionary in both fulfilling clinical needs and overcoming shortcomings of traditional procedures. Numerous preclinical studies have found that the use of rhBMP-2 induced exceptional bone regeneration within a short healing period [2–6]. Unfortunately, the use of rhBMP-2 is restricted to few indications (only maxillary sinus augmentation and extraction socket), due to its relatively high cost and potential complications such as extensive postoperative swelling and seroma formation [7, 8].

One possible way to limit these disadvantages is to reduce the amount of rhBMP-2 needed for the surgical intervention. One approach to consider is the simultaneous use of an agent—a so-called enhancer—that boosts the bone-regenerative effect. An ideal enhancer would increase the rhBMP-2 bioactivity, have no detrimental effects, be easy to apply clinically, and be inexpensive. Only a few studies have evaluated such enhancers [9–12]. In a preclinical study, N-methyl-2-pyrrolidone (NMP) was used to increase the flexibility of a barrier membrane [11]. Even though NMP was originally used as a plasticizer, enhanced bone regeneration was observed in rabbit calvarial defects. In further experiments, NMP treatment increased alkaline phosphatase activity and calcium deposition in the presence of rhBMP-2 at a lower-than-usual dose [10]. At the molecular level, NMP enhanced the kinase activity of the bone morphogenetic protein (BMP) receptor type I homodimer, which led to increases in Smad and p38 phosphorylation. Moreover, it was demonstrated that NMP inhibited osteoclast differentiation and function by blocking RANKL-induced AP-1 activation [13].

When rhBMP-2 is used for bone augmentation, the quantity of rhBMP-2 applied increases in proportion to the carrier material used to fill the defect area [14]. Considering that sinus augmentation requires a large amount of bone substitute material, overdosing of rhBMP-2 and high treatment costs are likely. Thus, the combined use of an enhancer and rhBMP-2 might offer several advantages for sinus augmentation procedures.

The aim of the present study was to determine whether or not NMP can decrease the dose of rhBMP-2 needed for localized bone regeneration in a rabbit sinus augmentation model.

## 2. Materials and Methods

### 2.1. Animals

Fifteen male New Zealand White rabbits (DooYeol Biotech, Seoul, Korea) weighing 2.5~3.0 kg were used in this study. Each animal was housed in its own cage under standard laboratory conditions. The animals were fed a pellet diet and had access to water ad libitum. The experiment was approved by the Institutional Animal Care and Use Committee of Yonsei Medical Center, Seoul, Korea (IACUC Approval number 2014-0364), and it was carried out between March 2014 and November 2014.

### 2.2. Preparation of Materials

The following materials were used for sinus augmentation: biphasic calcium phosphate consisting of 30% hydroxyapatite and 70%  ß-tricalcium phosphate (BCP; Bio-C, Cowellmedi, Busan, Korea), rhBMP-2 (Cowell BMP, Cowellmedi), and NMP (Sigma-Aldrich, St. Louis, MO, USA). These materials were combined in three treatment modalities for the experiment: BCP, BMP (rhBMP-2-coated BCP), and BMP/NMP (rhBMP-2-coated BCP soaked with NMP). RhBMP-2-coated BCP was produced as described earlier [15, 16]. Briefly, 0.025 mg of rhBMP-2 solution produced by* Escherichia coli* was mixed with 1 g of the BCP granules and lyophilized. The mixture was frozen on shelves at a temperature of –43°C for 3 hours, primarily dried in a condenser at –40°C, and then placed in a pressure chamber (5 mmHg) for 2 hours. Secondary drying was carried out by applying the following series at 5 mmHg: –20°C for 4 hours, –10°C for 4 hours, 0°C for 2 hours, and 20°C for 20 hours. For the BMP/NMP group, rhBMP-2-coated BCP particles were soaked in 0.2 mL of NMP (50 *μ*L/mL) for 5 minutes.

### 2.3. Surgical Intervention

Ketamine hydrochloride (Ketalar, Yuhan, Seoul, Korea) and xylazine (Rumpun, Bayer Korea, Seoul, Korea) were injected intramuscularly to induce general anesthesia. The surgical site was shaved and locally disinfected. Local anesthesia was induced using 2% lidocaine (lidocaine HCl, Huons, Seoul, Korea). Two experienced surgeons (Ui-Won Jung and Hyun-Chang Lim) performed the experimental surgery. A full-thickness flap was elevated after making a mid-sagittal incision over the nasal area. A circular reamer with a diameter of 5.5 mm (C-reamer, Neobiotech, Seoul, Korea) was used to access the sinus cavity under irrigation. The sinus bone wall was detached and the Schneiderian membrane was carefully elevated. Each sinus was then assigned to one of the following three modalities according to a computer-generated randomization list:

1. BMP/NMP (rhBMP-2-coated BCP particles soaked in NMP)
2. BMP (rhBMP-2-coated BCP particles)
3. BCP (BCP particles soaked in saline)

The amount of BCP used was 0.15 g in all groups. After sinus augmentation, the flap was sutured with 6-0 glyconate absorbable monofilament (Monosyn, B. Braun, Aesculap, PA, USA). Postoperative medication with 0.5 mg/kg ketorolac (Keromin, Hana Pharm, Seoul, Korea) and 5 mg/kg enrofloxacin (Baytril, Bayer Korea) was administered twice daily for 5 days postoperatively. All animals were sacrificed at 2 weeks after surgery using an overdose of anesthetic.

### 2.4. Histologic and Histomorphometric Analyses

The block sections were immersed in 5% formic acid for 14 days and then trimmed and embedded in paraffin. Coronal 5 *μ*m thick sections were made serially along the center of the window. The two central sections were stained with Masson's trichrome and hematoxylin-eosin. The specimens were examined using a binocular microscope (Leica DMLB, Leica Microsystems, Wetzlar, Germany). All images were captured and saved (cellSens Standard 1.11, Olympus Corporation, Center Valley, PA, USA) for histomorphometric evaluation.

**Figure 1:**
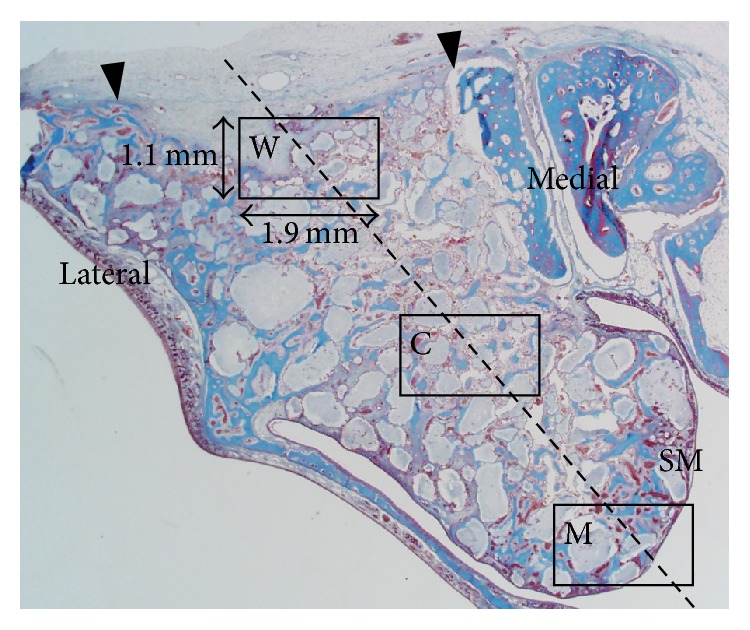
Histologic measurements of the augmented sinus. The total augmentation was defined as the area surrounded by the access window (arrowhead), lateral and medial sinus walls, and the Schneiderian membrane (SM). Three rectangular ROIs (1.1 mm × 1.9 mm) were established along the long axis of the augmentation: close to the access window (W), in the center (C), and near the Schneiderian membrane (M).

Descriptive histology was performed by analyzing the pattern of new bone (NB) formation, bone substitute degradation, and potentially adverse healing events. Histomorphometric measurement was performed in duplicate at 2-week intervals by a masked, experienced examiner (Hyun-Chang Lim) using an image processing program (Photoshop CS6, Adobe Systems, San Jose, CA, USA). The total augmented area (TA) encompassed a region bounded by the access window, the lateral sinus walls, and the Schneiderian membrane. Within the TA, the area of new bone (NB) and the areas of residual graft material (RM), and the area of nonmineralized tissue (NM) were calculated. Moreover, three rectangular regions of interest (ROIs) (1.1 mm × 1.9 mm) were designated within the sinus: close to the access window (W), in the center (C), and near the Schneiderian membrane (M) [17, 18]. NB, RM, and NM were calculated in these three ROIs (Figure 1).

#### 2.4.1. Primary Outcome

- NB within TA (NB_TA_).

#### 2.4.2. Secondary Outcomes

- RM and TA within TA (RM_TA_ and NM_TA_, resp.).
- NB, RM, and NM within the ROIs close to the bony window (W) and in the center (C) and membrane (M) regions (indicated using the subscripts “_ROI_W_”, “_ROI_C_”, and “_ROI_M_”, resp.).

### 2.5. Statistical Analysis

For sample size calculation, each sinus was regarded as a separate statistical unit. It was assumed that (i) the amount of NB formation expected for the control group (BCP) and the lower dose of rhBMP-2 (BMP) would be similar and that (ii) the addition of NMP to rhBMP-2 (BMP/NMP) would result in a larger amount of NB compared to BMP. The expected amount of NB was determined based on the findings of a previous study [3]. In order to achieve a power of 80% at an alpha level of 0.05, the calculated sample size required for the present study was 10 sinuses per group, and so 30 sinuses (from 15 rabbits) were prepared.

Histomorphometric data are presented as mean ± SD values, with each sinus serving as an individual statistical unit. The Shapiro-Wilk test was used to check that the data conformed to a normal distribution. One-way ANOVA or the Kruskal-Wallis test was used to assess statistical differences among three groups. Pairwise post hoc comparisons between BMP/NMP and BCP and between BMP and BCP were performed using Dunnett's test or the Mann–Whitney *U* test with Bonferroni correction (*P* = 0.05/2; SPSS 21.0, SPSS, Chicago, IL, USA). The cutoff for statistical significance was set at *P* < 0.05.

## 3. Results

### 3.1. Clinical Findings

There were no cases of sinus membrane perforation during the surgical interventions. Postoperative clinical healing was uneventful in all experimental animals, and no subsequent complications were observed during the healing period.

### 3.2. Descriptive Histology

**Figure 2:**
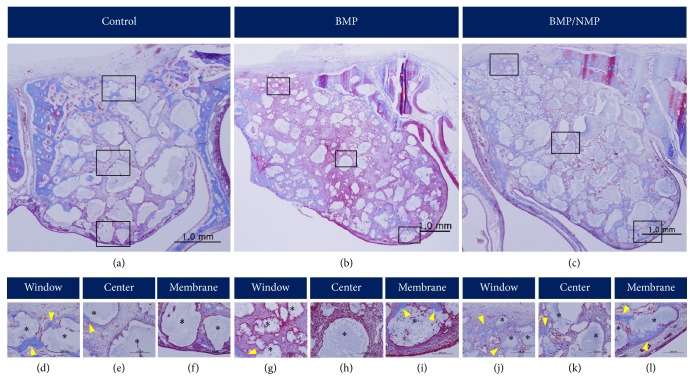
Histologic observations in the experimental groups (Masson's trichrome stain). ((a)–(c)) Overall views of the augmented sinus (original magnification, ×20). ((d)–(l)) High-magnification views of the boxed areas in panels (a)–(c) in each group (original magnification, ×200). Arrowheads, new bone; asterisks, residual bone material.

Partially inflated balloon-shaped augmentation was observed in all groups. The augmented area was surrounded by newly formed bone (NB) starting from the margins of the bony window, the lateral sinus walls, and the Schneiderian membrane. Bone substitute particles were observed within the entire augmented sinus and were partially surrounded by NB. Angiogenesis was observed throughout the specimens. Small blood vessels were observed even in the center of the augmented area (Figure 2).

**Figure 3:**
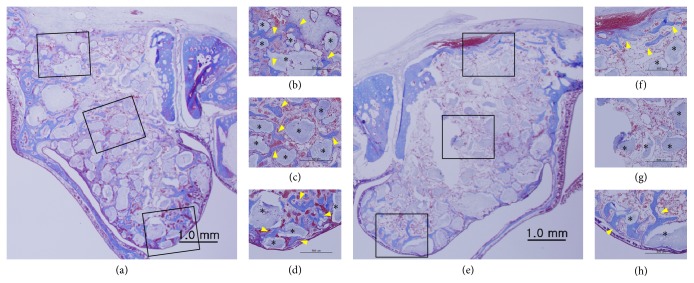
Histologic comparisons showing the least and greatest bone regeneration in the BMP/NMP group (Masson's trichrome stain). ((a), (e)) Overall views of the augmented sinus (original magnification, ×20). ((b), (f)) High-magnification views of the window region (original magnification, ×100). ((c), (g)) High-magnification views of the center region (original magnification, ×100). ((d), (h)) High-magnification view of the membrane region (original magnification, ×100). Arrowheads, new bone; asterisks, residual bone material.

The amount of NB formation in the center of the augmented sinus and in proximity to the Schneiderian membrane appeared to be greater in BMP/NMP than in BMP and BCP. Bone formation occurred predominantly near to native bone walls (i.e., bony window and lateral walls) in all groups. The residual graft material (RM) became more shattered in some of the NMP/BMP and BMP specimens. However, overall there was no remarkable difference in healing among the three groups. Moreover, all groups showed some variation in NB formation. Figure 2 shows histologic specimens representing the least bone formation (Figures 3(a)–3(d)) and greatest bone formation (Figures 3(e)–3(h)) in BMP/NMP.

### 3.3. Histomorphometric Analysis

All of the data obtained in the histomorphometric analysis are presented in Tables 1 and 2.

#### 3.3.1. Analysis within the Total Augmented Area

Newly formed bone area (NB_TA_) was greatest for BMP/NMP (1.94 ± 0.69 mm^2^), followed by BMP (1.50 ± 0.72 mm^2^) and BCP (1.28 ± 0.52 mm^2^). Similar trends were found for TA (20.05 ± 4.51 mm^2^ for BMP/NMP, 19.07 ± 4.42 mm^2^ for BMP, and 16.01 ± 3.01 mm^2^ for BCP), RM_TA_, and NM_TA_. There was no statistical difference in any of the above parameters among the three groups (*P* > 0.05).

**Figure 4:**
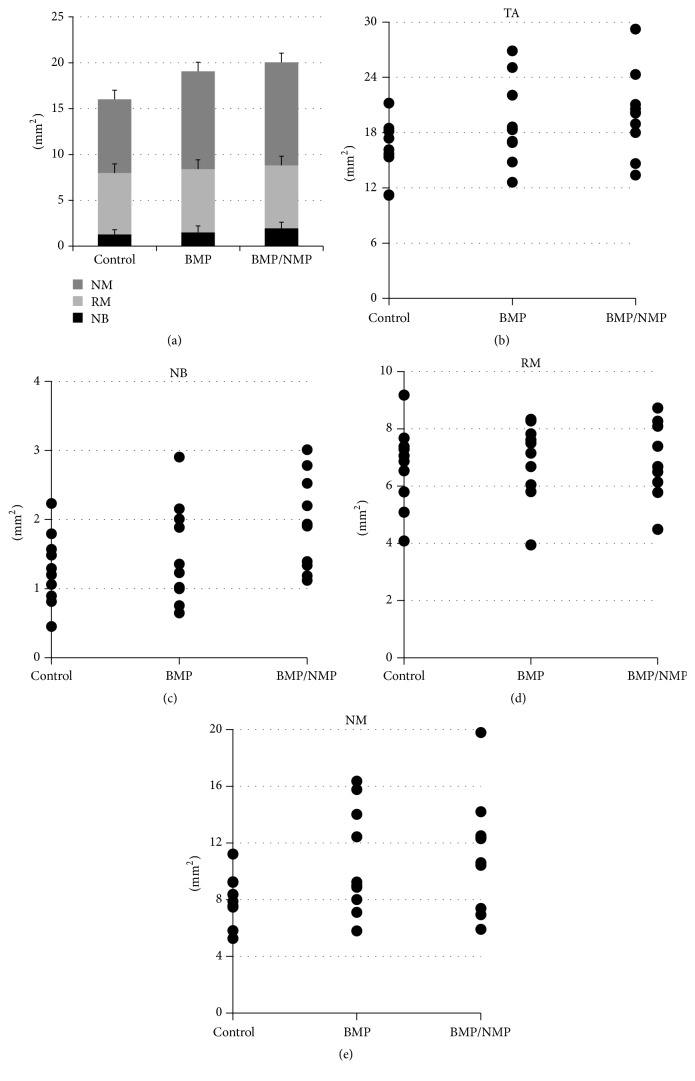
Comparisons of tissue components in the entire augmented sinus. (a) Bar graph. ((b)–(e)) Scatter plots of the area of total augmentation (TA), new bone (NB), residual material (RM), and nonmineralized tissue (NM), respectively.

Each measured value for TA, NB, RM, and NM demonstrated in the scattered plot that there was a large overlapping zone for the values among the three groups, indicating large variations between the specimens (Figure 4). This might explain why none of the statistical intergroup comparisons revealed any significant differences (see also Figure 2).

#### 3.3.2. Analyses within Regions of Interest

**Figure 5:**
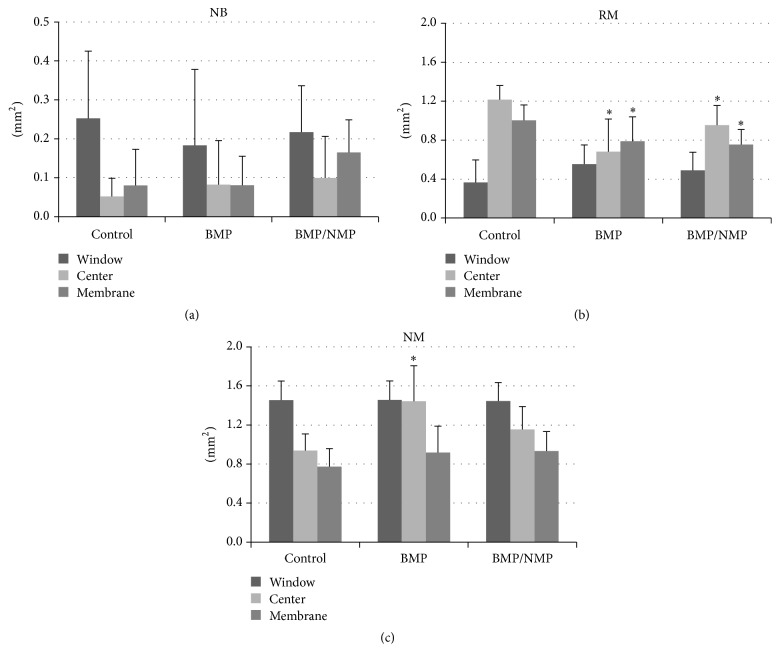
Findings of histomorphometric analyses in the regions of interest (ROIs). ((a)–(c)) Comparisons of the areas of new bone (NB), residual graft material (RM), and nonmineralized tissue (NM) in each region, respectively. Asterisks indicate statistical differences compared to the corresponding region for BCP.

Close to the bony window (W), the differences in newly formed bone (NB_ROI_W_) were small among the three treatment modalities (ranging from 0.18 ± 0.19 mm^2^ to 0.25 ± 0.17 mm^2^). The area of NB in the center (C) and membrane (M) regions was larger for BMP/NMP than for BMP and BCP. The area of NB within the C and M ROIs (NB_ROI_C_ and NB_ROI_M_) was 0.10 ± 0.11 mm^2^ and 0.17 ± 0.08 mm^2^, respectively, for BMP/NMP; the corresponding values were 0.08 ± 0.11 mm^2^ and 0.08 ± 0.07 mm^2^ for BMP and 0.05 ± 0.05 mm^2^ and 0.08 ± 0.09 mm^2^ for BCP (*P* > 0.05 for all comparisons) (Figure 5).

In W regions, the amount of bone substitute particles (RM_ROI_W_) was minimal, ranging from 0.37 ± 0.23 mm^2^ to 0.49 ± 0.19 mm^2^, and there were no significant differences between the three groups (*P* > 0.05). RM_ROI_C_ (1.22 ± 0.14 mm^2^) and RM_ROI_M_ (1.00 ± 0.16 mm^2^) were significantly larger for BCP than for BMP/NMP (0.95 ± 0.20 mm^2^ and *P* = 0.005, and 0.75 ± 0.15 mm^2^ and *P* = 0.014, resp.) and BMP (0.68 ± 0.33 mm^2^ and *P* < 0.001, and 0.79 ± 0.25 mm^2^ and *P* = 0.035).

The area of nonmineralized tissue (NM) was smallest in the ROI close to the Schneiderian membrane in all groups. NM_ROI_C_ was statistically larger for BMP than for BCP (*P* < 0.001). There were no other significant intergroup differences in NM_ROIs_ (*P* > 0.05).

## 4. Discussion

The present study investigated the effect of NMP as an enhancer for rhBMP-2 in a rabbit sinus augmentation model. It was found that (i) adding NMP to rhBMP-2 resulted in the largest area of newly formed bone within the entire augmented sinus and the greatest bone formation, even in regions further away from the access window, and (ii) there were large variations between the specimens, which was probably responsible for no significant differences being found between the treatment modalities.

Factors limiting the use of rhBMP-2 include its relatively high cost and postsurgical complications, which increase in proportion to the dose of rhBMP-2 that is applied to the defect. It was previously demonstrated that a small dose of rhBMP-2 significantly enhanced mineral deposition and the expression of bone formation markers in vitro when used together with NMP [11]. Moreover, the enhancement increased as the dose of rhBMP-2 was reduced, which suggests that a bone-regenerative effect could be maintained by decreasing the rhBMP-2 doses. This approach could therefore be expected to provide more predictable regeneration of large bone defects.

To date, few in vivo studies have tested the use of NMP for localized bone regeneration [11, 19], especially when using low-dose rhBMP-2 [10]. In calvarial defects of critical size in rabbits, it was demonstrated that the addition of NMP enhanced an ineffective rhBMP-2 dose (12 *μ*g), leading to significant bone regeneration compared to a control scaffold [10]. In the present study, a smaller dose (3.75 *μ*g) was applied to each sinus. This dose can be regarded as insufficient for bone regeneration in a rabbit sinus on the basis of previous results [16]. Following 2 weeks of healing after sinus augmentation, newly formed bone within the total augmented area (NB_TA_) was largest for BMP/NMP (1.5-fold larger than for BCP) and larger in the regions having a lower bone-regenerative potential (i.e., those in the center of the augmented sinus and close to the Schneiderian membrane) compared to other groups (twofold larger than for BCP). However, the increase in NB with the application of NMP, whether overall or in specific regions, did not reach statistical significance.

The data obtained in this pilot study imply that the addition of NMP to rhBMP-2 at an ineffective low dose might not predictably enhance bone regeneration. The average amount of newly formed bone in the BMP/NMP group appeared to be larger than in the control, but the degree of enhancement was inconsistent between the specimens. These outcomes might be explained by (i) large variations in healing characteristics between individual animals and (ii) the use of a suboptimal delivery system.

In the course of bone healing following rhBMP-2 delivery, in situ BMPs might act concomitantly with the exogenous rhBMP-2. While the amount of exogenous rhBMP-2 could have been equal in each sinus in the present study, the levels of in situ BMPs might vary between individual animals and sinuses. Considering that the effect of NMP might be proportional to the overall amount of BMP-2 from both sources, individual differences could be crucial when analyzing the effect of a low dose of exogenously delivered rhBMP-2. However, the variations in individual healing cannot be controlled. Ideally, the effect of bioactive agents should overcome individual differences and result in bone regeneration being consistently effective above a certain level. It has previously been reported that the individual healing potential might not be greatly influenced by applying NMP to a defect that is smaller than a critical size [11]. However, in the present study, larger defects (i.e., those in the sinus cavity) appeared to be affected to a greater extent by the individual healing potential. Specifically, large deviation in new bone formation of BCP clearly represented that individual healing was different (see Figure 4). This deviation was also demonstrated in BMP/NMP, and some specimens in BMP/NMP showed less new bone formation than BCP even though no surgical error and complication occurred. Such findings can be translated that the combination of low dose rhBMP-2 and NMP was not fully sufficient to enhance new bone formation especially in the animal having relatively low regenerative potential.

NMP is a water- and solvent-soluble molecule of low weight, which makes it difficult to deliver in a controlled and slow manner [10]. NMP incorporated in the membrane starts to be released immediately after being placed at the surgical site, and the membrane becomes stiff again [19]. Moreover, NMP is cleared from the body within a few hours [20, 21]. Similarly, a slow delivery system for NMP may be required for rhBMP-2 delivery. Alternatively, the periodic injection of NMP can also be considered because NMP is an injectable pharmaceutical excipient in FDA-approved formulations. A weekly injection of NMP was previously shown to produce antiosteoporotic activity in ovariectomized rats [22, 23]. However, those authors stated that the frequency and duration of the injections need be investigated further.

## 5. Conclusions

Using NMP as an adjunct to rhBMP-2-coated BCP increased bone regeneration in the augmented sinus cavity, specifically in the center of the augmentation and the region close to the Schneiderian membrane. However, the effects were inconsistent and did not result in significant differences compared to bone substitute alone. The individual healing potential may influence the effect of NMP, and so future studies should attempt to obtain consistent findings for bone regeneration.

## Figures and Tables

**Table 1:** Histomorphometric analysis in total augmentation (mean ± SD; mm^2^).

	BCP (*n* = 10)	BMP (*n* = 10)	BMP/NMP (*n* = 10)
TA	16.01 ± 3.01	19.07 ± 4.42	20.05 ± 4.51
NB_TA_	1.28 ± 0.52	1.50 ± 0.72	1.94 ± 0.69
RM_TA_	6.69 ± 1.43	6.92 ± 1.35	6.87 ± 1.28
NM_TA_	8.04 ± 1.71	10.66 ± 3.72	11.24 ± 4.07

No statistical difference among groups (*P* > 0.05); TA, total augmented area; NB_TA_, new bone area within TA; RM_TA_, residual graft material area within TA; NM_TA_, nonmineralized tissue area within TA.

**Table 2:** Histomorphometric analysis in the regions of interest (ROI, mean ± SD; mm^2^).

		BCP (*n* = 10)	BMP (*n* = 10)	BMP/NMP (*n* = 10)
NB_ROI__	W	0.25 ± 0.17	0.18 ± 0.19	0.22 ± 0.12
	C	0.05 ± 0.05	0.08 ± 0.11	0.10 ± 0.11
	M	0.08 ± 0.09	0.08 ± 0.07	0.17 ± 0.08

RM_ROI__	W	0.37 ± 0.23	0.55 ± 0.20	0.49 ± 0.19
	C	1.22 ± 0.14	0.68 ± 0.33^*∗*^	0.95 ± 0.20^*∗*^
	M	1.00 ± 0.16	0.79 ± 0.25^*∗*^	0.75 ± 0.15^*∗*^

NM_ROI__	W	1.45 ± 0.20	1.46 ± 0.19	1.44 ± 0.19
	C	0.94 ± 0.17	1.44 ± 0.36^*∗*^	1.15 ± 0.24
	M	0.77 ± 0.18	0.92 ± 0.27	0.93 ± 0.20

^*∗*^Statistically significant compared to BCP; for intergroup comparisons of NB_ROI_C_ and __M_, RM_ROI_C_, and NM_ROI_W_ and __C_, Kruskal-Wallis test and post hoc Mann–Whitney test using Bonferroni correction (*P* < 0.05/2) were used. For the rest of intergroup comparisons, one-way ANOVA with post hoc Dunnett's test was used (*P* < 0.05); W, the region adjacent to bony window; C, the center of the augmentation; M, the region adjacent to Schneiderian membrane; NB, new bone area; RM, residual graft material area; NM, nonmineralized tissue area.
